# Association between the fibrosis-4 index and carotid atherosclerosis in Chinese patients with type 2 diabetes mellitus: a cross-sectional study

**DOI:** 10.3389/fendo.2026.1856605

**Published:** 2026-07-13

**Authors:** Bo Huang, Yi-Rui Liang, Shi-Wei Li, Ya-Ning Li, Jing-Qiu Cui

**Affiliations:** Department of Endocrinology and Metabolism, Tianin Medical University General Hospital, Tianin, China

**Keywords:** carotid atherosclerosis, diabetic peripheral neuropathy, FIB-4, lower extremity arterial disease, restricted cubic spline, ROC analysis, type 2 diabetes mellitus

## Abstract

**Objective:**

This study aimed to investigate the association between the fibrosis-4 (FIB-4) index and diabetes-related vascular complications in patients with type 2 diabetes mellitus (T2DM). The primary outcome was carotid atherosclerosis (CAS), with lower extremity arterial disease (LEAD) and diabetic peripheral neuropathy (DPN) as secondary outcomes.

**Methods:**

This cross-sectional study included 1,045 patients with T2DM. Logistic regression models were applied to assess associations between FIB-4 and the risks of CAS, LEAD, and DPN. Restricted cubic spline (RCS) analyses were conducted to evaluate potential nonlinear relationships. Receiver operating characteristic (ROC) curves were used to determine the discriminatory performance of FIB-4 for each outcome. Subgroup analyses were performed to assess the robustness of the associations across different patient strata.

**Results:**

Higher FIB-4 levels were associated with increased odds of CAS after adjustment for potential confounders. In the fully adjusted model, with the lowest FIB-4 quartile as the reference, odds ratios for CAS in the second, third, and fourth quartiles were 1.16 (95% CI: 0.79–1.72), 1.75 (95% CI: 1.19–2.58), and 2.04 (95% CI: 1.39–3.00), respectively. RCS analysis demonstrated a nonlinear association between FIB-4 and CAS risk (P < 0.001). Limited discriminatory performance of FIB-4 for CAS was observed, with an area under the curve (AUC) of 0.579 and an optimal cutoff value of 0.94. Weaker but similar associations were identified for LEAD and DPN, with slightly higher discriminatory ability for LEAD (AUC = 0.587) compared to DPN (AUC = 0.532). Subgroup analyses generally supported the stability of these findings.

**Conclusion:**

Elevated FIB-4 was significantly associated with higher risks of CAS and LEAD in patients with T2DM, with the strongest association observed for CAS. The association between FIB-4 and DPN did not reach statistical significance after multivariable adjustment. FIB-4 may serve as a simple and practical adjunct marker for risk stratification of diabetes-related macrovascular complications, particularly CAS, in T2DM.

## 1 Introduction

Metabolic dysfunction-associated steatotic liver disease (MASLD) and type 2 diabetes mellitus (T2DM) frequently coexist within the same patient population, substantially increasing the risk of cardiovascular disease, which remains the leading cause of mortality in this group. Carotid atherosclerosis (CAS) represents a robust surrogate marker for future adverse cardiovascular events. In patients with MASLD, both hepatic steatosis and liver fibrosis have been strongly associated with CAS (1). Accumulating evidence indicates that liver fibrosis, rather than simple steatosis, may serve as an independent driver of atherosclerotic progression. Noninvasive fibrosis indices therefore provide practical tools for clinical risk stratification (2). Among these indices, the fibrosis-4 (FIB-4) index is widely recognized as a convenient and cost-effective marker of liver fibrosis.

Multiple cross-sectional and longitudinal studies have reported positive associations between FIB-4 levels and carotid intima-media thickness (cIMT) as well as carotid plaque formation in both general MASLD populations and individuals with T2DM. Kubotsu et al. (3) demonstrated a significant correlation between FIB-4 and maximum cIMT in patients with MASLD, with FIB-4 independently associated with significant CAS in this cohort. Su et al. (4) further confirmed this relationship in a large Chinese population, showing that elevated FIB-4 remained independently associated with increased CAS risk in NAFLD, even after adjustment for traditional cardiovascular risk factors. In patients with T2DM, Lee et al. (5) reported an independent association between FIB-4-based liver fibrosis and incident CAS over a 6- to 8-year follow-up period. Lu et al. (6) extended these observations to MASLD, reporting that higher FIB-4 scores correlated with more severe coronary artery disease and CAS.

Despite consistent findings across Western and East Asian cohorts, cross-sectional evidence specifically addressing the association between FIB-4 and CAS in Chinese patients with T2DM remains limited. In addition, the relationships between FIB-4 and other diabetes-related complications, including lower extremity arterial disease (LEAD) and diabetic peripheral neuropathy (DPN), have not been sufficiently characterized. Accordingly, the present study investigated the association between FIB-4 and CAS in a Chinese T2DM population, with LEAD and DPN evaluated as secondary outcomes. The potential utility of FIB-4 as a practical marker for risk stratification of subclinical atherosclerosis in this high-risk population was also assessed.

## 2 Materials and methods

### 2.1 Study population

The study population consisted of patients with T2DM treated in the Department of Endocrinology and Metabolism at Tianjin Medical University General Hospital between 2021 and 2024. Inclusion criteria were as follows (1): age 45–80 years; (2) a confirmed diagnosis of T2DM according to the 2019 Standards of Medical Care in Diabetes established by the American Diabetes Association; and (3) availability of complete carotid and lower extremity arterial ultrasonography data, together with complete assessments for DPN.

**Figure 1:**
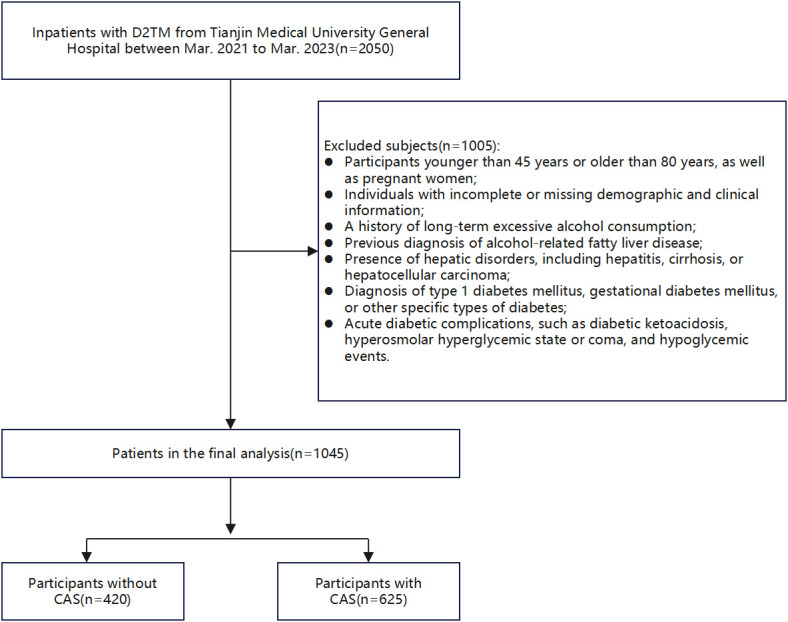
Flowchart of the study population selection process.

Exclusion criteria included: (1) incomplete or missing demographic or clinical data; (2) a history of long-term excessive alcohol consumption; (3) a prior diagnosis of alcohol-related fatty liver disease; (4) hepatic diseases, including hepatitis, cirrhosis, or hepatocellular carcinoma; (5) gestational diabetes mellitus, type 1 diabetes mellitus, or other specific types of diabetes; and (6) acute diabetic complications, including hypoglycemic episodes, hyperosmolar hyperglycemic state or coma, and diabetic ketoacidosis. A total of 1,045 participants were ultimately included in the final analysis (**Figure 1**).

### 2.2 Covariates

On the morning following admission, fasting venous blood samples were obtained from all participants after an overnight fast. Laboratory measurements were performed in the hospital’s clinical laboratory using standard methods, including fasting blood glucose (FBG), low-density lipoprotein cholesterol (LDL-C), high-density lipoprotein cholesterol (HDL-C), total cholesterol (TC), triglycerides (TG), glycated hemoglobin A1c (HbA1c), alanine aminotransferase (ALT), aspartate aminotransferase (AST), gamma-glutamyl transferase (GGT), creatinine (Cr), platelet count (PLT), albumin, and serum uric acid (SUA).

Body mass index (BMI) was calculated as weight in kilograms divided by height in meters squared (8). Hypertension was defined as a self-reported history of hypertension, or a systolic blood pressure (SBP) ≥ 140 mmHg, or a diastolic blood pressure (DBP) ≥ 90 mmHg (9). The FIB-4 index was calculated using the following formula (7–9):


$$FIB-4\;index=\frac{age(years)\times AST(IU/L)}{PLT\;count(10^{9}/L)\times \sqrt{ALT(IU/L)}}$$

### 2.3 Definitions of CAS, LEAD, and DPN

Both CAS and LEAD were evaluated using ultrasonography. Intima-media thickness was defined as the distance between the leading edges of the first and second echogenic lines. Plaque was defined as a focal structure protruding into the arterial lumen and meeting at least one of the following criteria: intima-media thickness ≥ 0.5 mm, thickness ≥ 50% greater than that of the adjacent vessel wall, or localized intima-media thickness > 1.5 mm (10).

DPN was assessed using the Michigan Neuropathy Screening Instrument (MNSI), comprising a 15-item questionnaire and a lower-extremity physical examination (11). The physical examination evaluated foot appearance, foot ulcers, vibration perception, and ankle reflexes, with a maximum score of 8 points. DPN was defined as an examination score ≥ 2.5 points (12). An abnormal 10-g monofilament test was defined as fewer than seven correctly perceived tactile points on both feet. All examinations were performed by trained nurses (13).

### 2.4 Statistical analysis

Continuous variables were summarized according to their distribution: normally distributed variables were expressed as mean ± standard deviation, whereas non-normally distributed variables were presented as median (interquartile range, IQR; 25th–75th percentiles). Categorical variables were expressed as counts and percentages. Between-group comparisons across the four FIB-4 quartiles were performed using one-way analysis of variance for normally distributed variables, the Kruskal–Wallis H test for non-normally distributed variables, and the chi-square test for categorical variables. Participants were stratified into four groups according to FIB-4 quartiles: Q1 (FIB-4 ≤ 0.70), Q2 (0.70 < FIB-4 ≤ 1.04), Q3 (1.04 < FIB-4 ≤ 1.75), and Q4 (FIB-4 > 1.75).

Logistic regression models were used to evaluate associations between FIB-4 and CAS, LEAD, and DPN. To reduce potential multicollinearity among lipid variables, LDL-C, HDL-C, and TC were excluded simultaneously from the fully adjusted model, with TG retained as the representative lipid parameter. Multicollinearity was assessed using the variance inflation factor (VIF), with results presented in **Supplementary Table 1**. Associations were reported as odds ratios (ORs) with 95% confidence intervals (CIs). Receiver operating characteristic (ROC) curve analyses were performed to evaluate the discriminatory performance of FIB-4, with area under the curve (AUC), optimal cutoff values, sensitivity, specificity, and Youden index calculated accordingly. All tests were two-sided, with P < 0.05 considered statistically significant. Statistical analyses were conducted using SPSS version 26.0, and forest plots were generated using R version 4.3.1.

## 3 Results

### 3.1 Baseline characteristics of the study population

**Table 1** summarizes the baseline characteristics of participants stratified by FIB-4 quartiles. No significant between-group differences were observed for sex distribution, BMI, smoking status, drinking status, SBP, hypertension, FBG, SUA, Cr, HbA1c, TC, TG, HDL-C, or LDL-C (all P > 0.05).

**Table 1:** Characteristics of patients categorized by FIB-4 quartiles.

Parameters	Q1 (n=263)	Q2 (n=256)	Q3 (n=265)	Q4 (n=261)	P value
Male, n (%)	135 (51.3%)	148 (57.8%)	153 (57.7%)	160 (61.3%)	0.136
Age (years)	52 (45, 60)	58 (51, 63)*	60 (53, 66)*#	62 (56, 66)*#	<0.001
BMI (kg/m^2^)	26.0(23.6,29.3)	25.7(23.5,28.1)	25.5(23.7,27.8)	24.9(23.4,27.7)	0.072
Smoking,n (%)	85 (32.3%)	98 (38.3%)	93 (35.1%)	110 (42.1%)	0.110
Drinking,n (%)	75 (28.5%)	92 (35.9%)	87 (32.8%)	99 (37.9%)	0.116
SBP (mmHg)	132 (121, 146)	134 (124, 145)	132(123, 143)	133(123, 145)	0.640
DBP (mmHg)	83.9 ± 12.1	83.6 ± 10.4	81.1 ± 11.1	82.2 ± 12.1	0.017
HN, n(%)	137 (52.1%)	133 (52.0%)	153 (57.7%)	138 (52.9%)	0.494
DPN, n (%)	56 (21.3%)	83 (32.4%)*	71 (26.8%)	75 (28.7%)	0.037
CAS, n (%)	125 (47.5%)	157 (61.3%)*	175 (66.0%)*	168 (64.4%)*	<0.001
LEAD, n (%)	150 (57.0%)	177 (69.1%)*	195 (73.6%)*	196 (75.1%)*	<0.001
FBG (mmol/L)	7.3 (5.9, 9.7)	7.1 (5.2, 9.8)	6.8 (5.6, 9.3)	7.0 (5.6, 9.2)	0.399
SUA (μmol/L)	315(246,381)	329 (267, 399)	346(265, 412)	330(258, 395)	0.054
Cr (μmol/L)	59 (48, 72)	62 (50, 71)	61 (50, 73)	62 (53, 73)	0.056
ALT (U/L)	34 (21, 65)	21 (16, 32)*	17 (13, 24)*#	16 (11, 23)*#	<0.001
AST (U/L)	14 (12, 17)	17 (14, 20)*	19 (16, 24)*#	32(24,49)*#&	<0.001
GGT (U/L)	35 (21, 68)	27 (18, 49)*	22 (15, 34)*#	22 (15, 36)*#	<0.001
HbA1c (%)	8.19 ± 2.10	8.41 ± 2.03	8.39 ± 2.12	8.55 ± 2.04	0.060
TC (mmol/L)	4.65(4.08,5.50)	4.66 (4.02,5.47)	4.80 (3.84,5.59)	4.76 (4.04,5.62)	0.739
TG (mmol/L)	1.67 (1.14,2.44)	1.60 (1.13,2.51)	1.67 (1.21, 2.43)	1.68 (1.19, 2.49)	0.831
HDL (mmol/L)	1.02 (0.88,1.25)	1.04 (0.89,1.22)	1.02 (0.88, 1.22)	1.04 (0.88, 1.19)	0.983
LDL (mmol/L)	2.84 (2.24,3.46)	2.76 (2.22,3.42)	2.85 (2.24, 3.51)	2.91 (2.34, 3.52)	0.697

Data are presented as median (interquartile range) or mean ± SD for continuous variables and n (%) for categorical variables. Continuous variables were compared using the Kruskal–Wallis test, except DBP and HbA1c (one-way ANOVA); categorical variables were compared using the chi-square test. *Post-hoc* pairwise comparisons: *p < 0.05 vs. Q1; #p < 0.05 vs. Q2; &p < 0.05 vs. Q3 (Mann–Whitney U or chi-square test with Bonferroni correction). BMI, body mass index; HN, hypertension; DPN, diabetic sensorimotor polyneuropathy; CAS, carotid atherosclerosis; LEAD, lower extremity arterial disease; SBP, systolic blood pressure; DBP, diastolic blood pressure; FBG, fasting blood glucose; SUA, serum uric acid; Cr, creatinine; ALT, alanine aminotransferase; AST, aspartate aminotransferase; GGT, glutamyl transpeptidase; HbA1c, glycated hemoglobin A1c; TC, total cholesterol; TG, triglyceride; HDL, high-density lipoprotein; LDL, low-density lipoprotein.

Age increased progressively across ascending FIB-4 quartiles (P < 0.001), whereas DBP showed a slight decline in higher quartiles (P = 0.017). The prevalence of DPN, CAS, and LEAD increased significantly across FIB-4 quartiles (all P < 0.05), indicating a higher burden of vascular and neuropathic complications among individuals with elevated FIB-4 levels.

**Figure 2:**
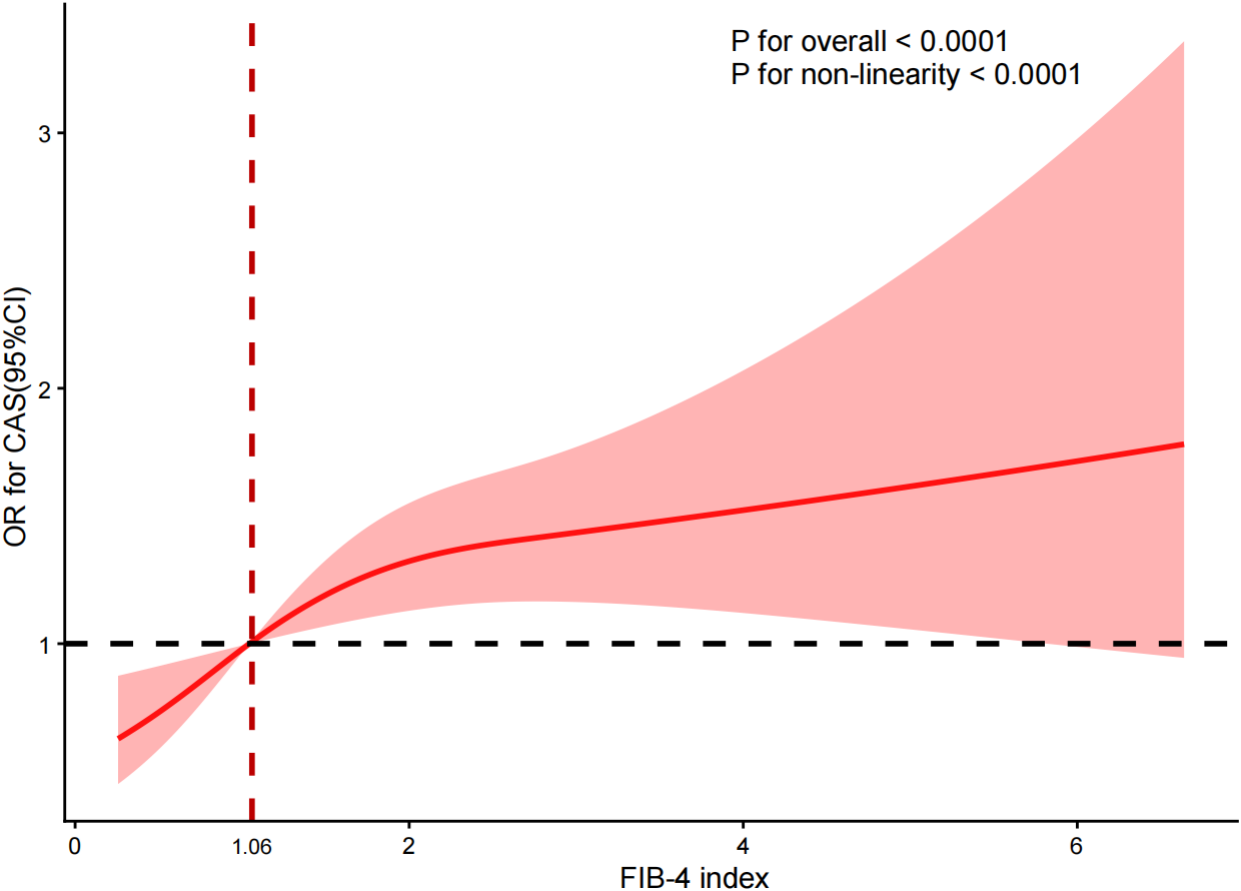
Prevalence of carotid atherosclerosis according to FIB-4 quartiles.

**Figure 2** depicts the prevalence of CAS across FIB-4 quartiles. The proportion of patients with CAS increased from 47.53% in Q1 to 61.33% in Q2 and 66.04% in Q3, remaining elevated at 64.37% in Q4. Conversely, the proportion without CAS decreased from 52.47% in Q1 to 35.63% in Q4. These patterns suggest an overall positive association between FIB-4 and CAS, with a tendency toward plateauing at higher FIB-4 levels.

### 3.2 Correlations between FIB-4 and clinical parameters

**Table 2** shows that FIB-4 was positively correlated with age (r = 0.325, P < 0.001), Cr (r = 0.081, P = 0.009), AST (r = 0.669, P < 0.001), and HbA1c (r = 0.071, P = 0.022), and negatively correlated with BMI (r = −0.074, P = 0.016), DBP (r = −0.070, P = 0.023), ALT (r = −0.462, P < 0.001), and GGT (r = −0.246, P < 0.001). No significant correlations were observed between FIB-4 and SBP, FBG, SUA, or lipid parameters (all P > 0.05).

**Table 2:** Relationships between FIB-4 and other factors.

Factors	R	P value
Age (years)	0.325	<0.001
BMI (kg/m^2^)	-0.074	0.016
SBP (mmHg)	-0.002	0.961
DBP (mmHg)	-0.070	0.023
FBG (mmol/L)	-0.016	0.612
SUA (μmol/L)	0.042	0.176
Cr (μmol/L)	0.081	0.009
ALT (U/L)	-0.462	<0.001
AST (U/L)	0.669	<0.001
GGT (U/L)	-0.246	<0.001
HbA1c (%)	0.071	0.022
TC (mmol/L)	0.020	0.525
TG (mmol/L)	0.023	0.449
HDL (mmol/L)	-0.003	0.928
LDL (mmol/L)	0.018	0.560

Spearman’s rank correlation coefficients (r) between FIB-4 and clinical/biochemical parameters are presented. BMI, body mass index; SBP, systolic blood pressure; DBP, diastolic blood pressure; FBG, fasting blood glucose; SUA, serum uric acid; Cr, creatinine; ALT, alanine aminotransferase; AST, aspartate aminotransferase; GGT, glutamyl transpeptidase; HbA1c, glycated hemoglobin A1c; TC, total cholesterol; TG, triglyceride; HDL, high-density lipoprotein; LDL, low-density lipoprotein.

### 3.3 Associations of FIB-4 quartiles with CAS

Multivariable logistic regression analyses assessed the association between FIB-4 quartiles and CAS risk, with Q1 as the reference group (**Table 3**). In the unadjusted model (Model 1), significantly higher odds of CAS were observed in Q3 and Q4 compared to Q1, with ORs of 1.89 (95% CI: 1.30–2.75, P < 0.001) and 2.18 (95% CI: 1.50–3.17, P < 0.001), respectively, whereas Q2 showed no significant association (OR = 1.22, 95% CI: 0.83–1.78, P = 0.311). After adjustment for sex, smoking status, and drinking status in Model 2, the associations remained largely unchanged. In the fully adjusted model (Model 3), effect estimates were slightly attenuated, but the associations for Q3 and Q4 remained statistically significant, indicating an independent association between higher FIB-4 levels and increased CAS risk.

**Table 3:** Association between FIB-4 quartiles and CAS.

		Reference (Q1)	Q2 OR (95% CI)	P	Q3 OR (95% CI)	P	Q4 OR (95% CI)	P
CAS	Model 1	**1**	1.22 (0.83–1.78)	0.311	1.89 (1.30–2.75)	<0.001	2.18 (1.50–3.17)	<0.001
	Model 2	**1**	1.21 (0.82–1.77)	0.333	1.87 (1.28–2.72)	0.001	2.13 (1.46–3.10)	<0.001
	Model 3	**1**	1.16 (0.79–1.72)	0.448	1.75 (1.19–2.58)	0.004	2.04 (1.39–3.00)	<0.001

Results are expressed as odds ratio (OR) with 95% confidence interval (CI) from binary logistic regression. Q1 is the reference group. Model 1: unadjusted. Model 2: adjusted for sex, smoking, and alcohol consumption. Model 3: adjusted for sex, smoking, alcohol consumption, BMI, FBG, GGT, HbA1c, TG, SUA, Cr, and HN. CAS, carotid atherosclerosis; BMI, body mass index; FBG, fasting blood glucose; GGT, glutamyl transpeptidase; HbA1c, glycated hemoglobin A1c; TG, triglyceride; SUA, serum uric acid; Cr, creatinine; HN, hypertension.

For secondary outcomes, a similar but weaker pattern was observed for LEAD, whereas no significant association with DPN was identified after multivariable adjustment (**Supplementary Table 2**). In the fully adjusted model, ORs for DPN were 1.43 (95% CI: 0.91–2.24, P = 0.123), 1.17 (95% CI: 0.75–1.84, P = 0.484), and 1.37 (95% CI: 0.88–2.12, P = 0.164) for Q2, Q3, and Q4, respectively, compared to Q1. For LEAD, higher FIB-4 quartiles were associated with increased odds of disease. Compared to Q1, fully adjusted ORs were 1.09 (95% CI: 0.72–1.63, P = 0.688) for Q2, 1.60 (95% CI: 1.06–2.41, P = 0.024) for Q3, and 1.88 (95% CI: 1.24–2.84, P = 0.003) for Q4.

### 3.4 Nonlinear association between FIB-4 and CAS: RCS analysis

**Figure 3:**
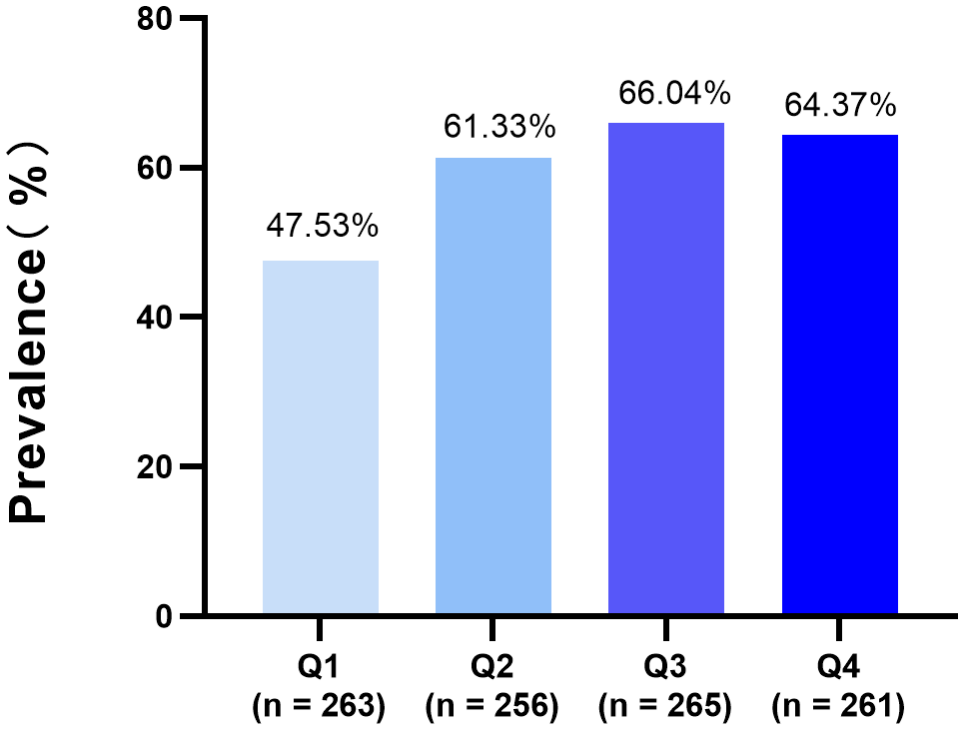
Restricted cubic spline analysis of the association between FIB-4 and the odds of carotid atherosclerosis. CI, confidence interval; RCS, restricted cubic spline.

**Figure 3** illustrates the association between FIB-4 and CAS risk based on restricted cubic spline (RCS) analysis. Significant overall and non-linear associations were observed between FIB-4 and CAS risk (P for overall < 0.0001; P for non-linearity < 0.0001). The odds of CAS increased steeply at lower FIB-4 levels, followed by a more gradual upward trajectory. The spline curve crossed the reference line of OR = 1 at an FIB-4 value of approximately 1.06; however, this value should be interpreted as a model-derived reference point rather than a clinically validated threshold.

For secondary outcomes, distinct patterns were observed (**Supplementary Figure 1**). A significant overall association between FIB-4 and LEAD risk was identified (P for overall = 0.0012), whereas the non-linear component did not reach statistical significance (P for non-linearity = 0.0853). The spline curve suggested a generally increasing trend in LEAD risk with higher FIB-4 levels, crossing OR = 1 at approximately 1.0589. In contrast, neither the overall nor the non-linear association between FIB-4 and DPN was statistically significant (P for overall = 0.2620; P for non-linearity = 0.1555). Although the DPN curve showed fluctuations across the range of FIB-4 values, wide CIs largely overlapped with OR = 1, indicating substantial uncertainty.

### 3.5 Diagnostic performance of FIB-4 for CAS

**Figure 4:**
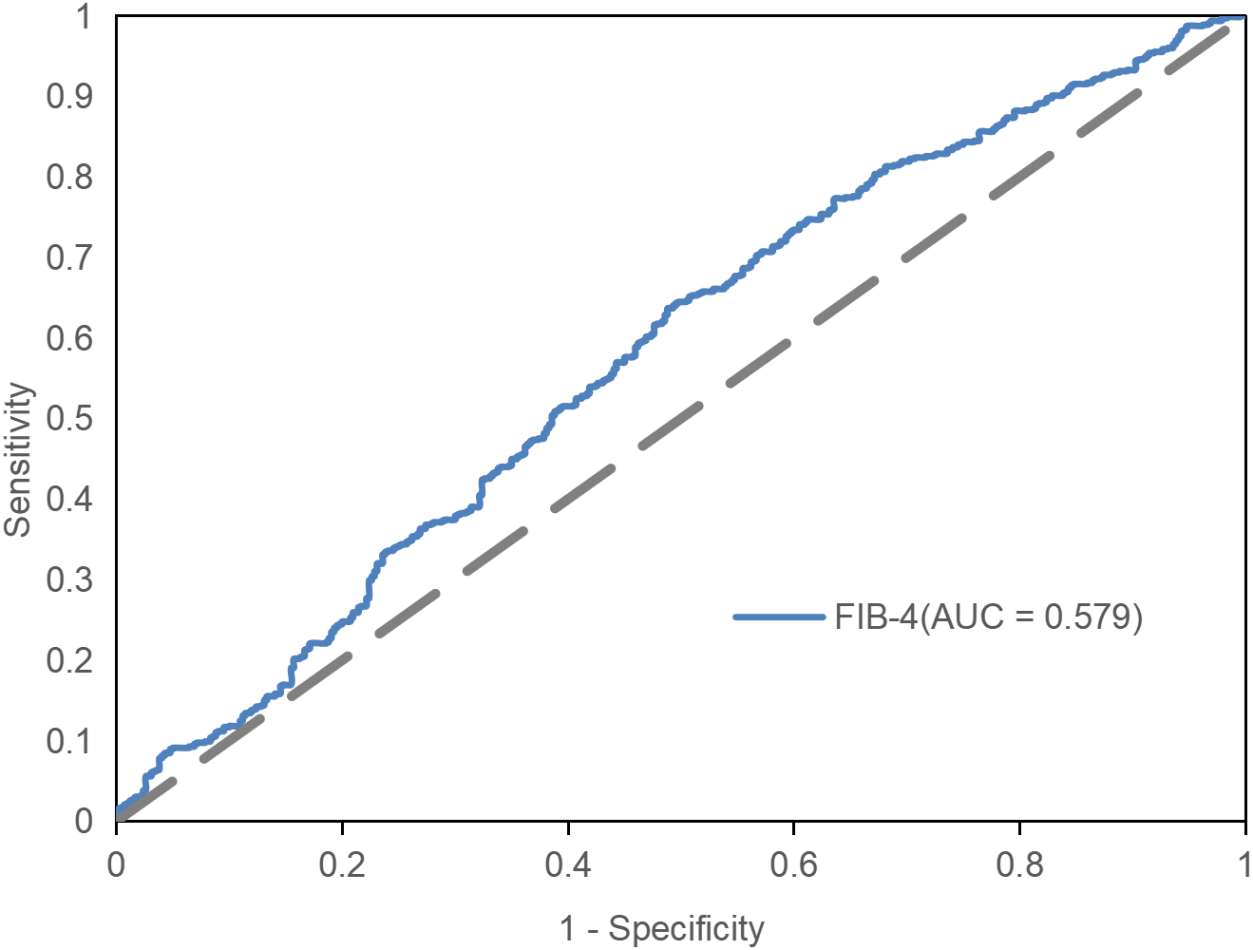
Receiver operating characteristic curve of FIB-4 for identifying carotid atherosclerosis.

ROC analysis demonstrated limited discriminatory performance of FIB-4 for CAS. As shown in **Figure 4**, **Table 4**, the AUC was 0.579 (95% CI: 0.546–0.616). The optimal cutoff value was 0.94, corresponding to a sensitivity of 63.7% and a specificity of 51.2%.

**Table 4:** Diagnostic performance of FIB-4 for carotid atherosclerosis.

Outcome	AUC (95% CI)	Cut-off value	Sensitivity (%)	Specificity (%)	PPV (%)	NPV (%)	Youden index
CAS	0.579 (0.546–0.616)	0.94	63.7	51.2	66.0	48.6	0.149

The optimal cut-off value was determined using the maximum Youden index from receiver operating characteristic (ROC) analysis. The 95% confidence interval for AUC was derived by 1,000-iteration bootstrap resampling.

For secondary outcomes, similarly limited performance was observed. The AUC was 0.587 (95% CI: 0.552–0.626) for LEAD and 0.532 (95% CI: 0.492–0.568) for DPN (**Supplementary Figure 2**, **Supplementary Table 3**). Overall, these results indicate limited discriminatory capacity of FIB-4 for CAS, LEAD, and DPN, precluding its use as a stand-alone diagnostic tool.

### 3.6 Subgroup analyses of the association between FIB-4 and CAS

**Figure 5:**
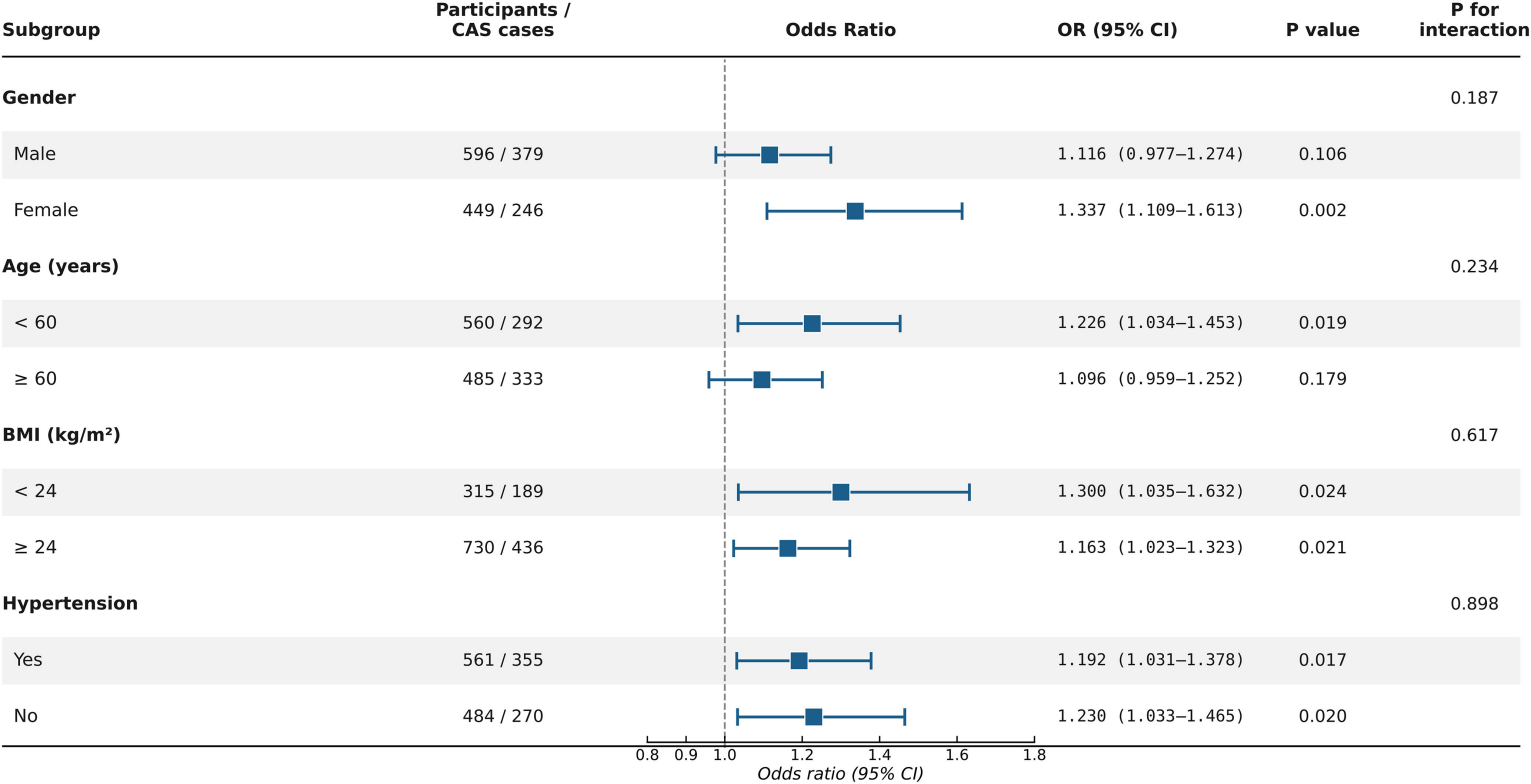
Subgroup analyses of the association between FIB-4 and carotid atherosclerosis.

Subgroup analyses indicated that higher FIB-4 levels were associated with increased CAS risk across most strata (**Figure 5**). This association reached statistical significance in women, participants aged < 60 years, both BMI subgroups, and individuals with or without hypertension. However, significance was not observed in men or participants aged ≥ 60 years. No significant interactions were detected across subgroups stratified by sex, age, BMI, or hypertension status, suggesting a generally consistent association between FIB-4 and CAS.

For secondary outcomes, subgroup consistency was less evident. For LEAD, higher FIB-4 levels were significantly associated with increased risk in both sexes, in participants with BMI ≥ 24 kg/m^2^, and in those with hypertension, whereas associations were not significant in either age subgroup, in participants with BMI < 24 kg/m^2^, or in those without hypertension. No significant interactions were observed across any subgroup stratifications. In contrast, for DPN, no significant associations were identified in any subgroup, and no significant interactions were detected across sex, age, BMI, or hypertension strata (**Supplementary Figures 3**, **S4**).

## 4 Discussion

This study systematically evaluated the associations between FIB-4 and diabetes-related complications in patients with T2DM. Among the examined outcomes, elevated FIB-4 showed the strongest and most consistent independent association with CAS risk. Both quartile-based logistic regression and RCS analyses demonstrated a dose–response pattern and a significant non-linear association between FIB-4 and CAS. However, the discriminatory performance of FIB-4 was limited, as reflected by relatively low AUC, sensitivity, and specificity. Accordingly, FIB-4 should not be considered a stand-alone diagnostic or screening tool for CAS. Instead, it may serve as a simple and inexpensive adjunct marker for risk stratification, helping to identify patients with T2DM who may benefit from further vascular evaluation using established imaging modalities, such as carotid ultrasonography.

In this cross-sectional study, higher FIB-4 levels were associated with an increased prevalence of CAS in patients with T2DM, and the association remained significant after adjustment for multiple clinical and metabolic covariates. However, causal inference cannot be established due to the cross-sectional design. In addition, because FIB-4 is derived from age, AST, ALT, and PLT, its association with CAS may reflect not only hepatic fibrosis but also the combined influence of aging, liver enzyme alterations, platelet-related factors, and overall metabolic burden.

The use of FIB-4 quartiles and RCS analysis enabled assessment of graded and dose–response relationships between FIB-4 and diabetes-related complications. Although categorization of FIB-4 may result in some loss of information, the persistence of associations after multivariable adjustment suggests potential incremental value in identifying patients with T2DM at higher risk of CAS. Compared to CAS, the weaker associations observed for LEAD and DPN may be attributable to differences in outcome assessment methods, disease characteristics, and statistical power. While CAS and LEAD were assessed by ultrasonography, DPN was evaluated using the MNSI; therefore, comparisons across outcomes should be interpreted with caution. Overall, the findings suggest that FIB-4 may be more closely related to macrovascular atherosclerotic changes than to neuropathic complications in this population.

Atherosclerosis is a progressive process that may develop over many years before clinical cardiovascular events occur, with CAS serving as an important marker of subclinical vascular disease and a predictor of future cardiovascular risk (14, 15). These findings are broadly consistent with previous studies reporting cross-sectional associations between NAFLD and subclinical atherosclerosis (16–20), as well as evidence suggesting that FIB-4 has prognostic value beyond liver fibrosis, extending to cardiovascular and all-cause outcomes (21). Prior studies have linked higher FIB-4 levels to coronary artery disease in patients with MASLD and to increased cIMT in individuals with metabolic disorders (22). In addition, a Korean longitudinal cohort study reported that hepatic steatosis and liver fibrosis were associated with accelerated CAS progression in patients with T2DM over a 6- to 8-year follow-up period (5). In contrast, the present study focused specifically on CAS in a T2DM population and applied multiple complementary analytical approaches to characterize the dose–response relationship, discriminatory performance, and subgroup consistency. Nevertheless, given the cross-sectional design, the observed associations should be interpreted as hypothesis-generating rather than causal.

The association between FIB-4 and CAS may be partially explained by shared metabolic and inflammatory pathways linking hepatic fibrosis to subclinical atherosclerosis in T2DM. MASLD is highly prevalent among patients with T2DM, and both conditions share key pathogenic mechanisms involved in atherosclerosis, including insulin resistance, dyslipidemia, and chronic low-grade inflammation (22). Accordingly, FIB-4 may reflect not only the extent of liver fibrosis but also the overall burden of metabolic dysfunction, inflammation, oxidative stress, platelet-mediated vascular injury, and endothelial dysfunction. A fibrotic liver may further amplify a proinflammatory and proatherogenic milieu, thereby promoting plaque formation and vascular remodeling. Insulin resistance is likely a central shared mechanism; however, as HOMA-IR and HOMA-β were not assessed in this study, this interpretation remains speculative and requires confirmation in future studies incorporating fasting insulin or C-peptide measurements. Emerging evidence suggests that integration of metabolic profiling, inflammatory status, and vascular assessment may improve characterization of subclinical atherosclerosis in high-risk metabolic populations (23, 24). In this context, FIB-4 may be more appropriately considered an adjunctive risk marker within a comprehensive metabolic–vascular assessment framework rather than an independent diagnostic indicator.

This study has several limitations. First, the cross-sectional design precludes causal inference. Second, the single-center setting may limit generalizability. Third, despite adjustment for multiple potential confounders, residual confounding from unmeasured factors such as lifestyle behaviors and medication use cannot be excluded. Fourth, FIB-4 is an indirect surrogate of liver fibrosis, and no direct assessment using imaging modalities or biopsy was performed. In addition, CAS, LEAD, and DPN were evaluated using different diagnostic approaches, which may limit direct comparability across outcomes, although each method is widely accepted for its respective condition. Finally, the absence of fasting insulin data prevented calculation of HOMA-IR and HOMA-β, thereby limiting mechanistic interpretation. Future prospective multicenter studies incorporating direct liver fibrosis assessment, more comprehensive vascular imaging, and standardized neurological evaluation are warranted to validate these findings and clarify underlying mechanisms.

## 5 Conclusion

In patients with T2DM, higher FIB-4 levels were significantly associated with an increased risk of CAS, with a clear dose–response relationship across quartiles and a non-linear association between FIB-4 and CAS risk. The association remained significant after multivariable adjustment and was generally consistent across subgroups, although variation by age and hypertension status was observed. Despite limited discriminatory performance for CAS identification, FIB-4 may still serve as a simple and readily available adjunctive marker for vascular risk stratification. Prospective studies are required to confirm these findings and further elucidate the biological mechanisms linking FIB-4 to macrovascular complications in T2DM.

## Data Availability

The raw data supporting the conclusions of this article will be made available by the authors, without undue reservation.
