# Supplementary material for: Association between the fibrosis-4 index and carotid atherosclerosis in Chinese patients with type 2 diabetes mellitus: a cross-sectional study
Source: Front Endocrinol (Lausanne). 2026 Jul 13;17:1856605. doi: 10.3389/fendo.2026.1856605 (PMC13402159; doi:10.3389/fendo.2026.1856605)
Supplement: Supplementary file 1 [file SupplementaryFile1.docx]

**Supplementary Table S1. Multicollinearity diagnostics of variables included in the multivariable logistic regression models**

| Variable | Tolerance | VIF |
| --- | --- | --- |
| Sex | 0.559 | 1.788 |
| Smoking history | 0.611 | 1.635 |
| Drinking history | 0.591 | 1.693 |
| BMI, kg/m² | 0.962 | 1.040 |
| FBG | 0.984 | 1.016 |
| GGT | 0.954 | 1.048 |
| HbA1c | 0.983 | 1.018 |
| TG | 0.990 | 1.010 |
| SUA | 0.989 | 1.011 |
| Cr | 0.848 | 1.180 |
| Hypertension | 0.937 | 1.067 |
| FIB-4 Q2 | 0.542 | 1.844 |
| FIB-4 Q3 | 0.512 | 1.952 |
| FIB-4 Q4 | 0.510 | 1.962 |

Note: VIF, variance inflation factor; FIB-4, fibrosis-4 index; BMI, body mass index; FBG, fasting blood glucose; GGT, γ-glutamyl transferase; HbA1c, glycated hemoglobin; TG, triglycerides; SUA, serum uric acid; Cr, creatinine. FIB-4 quartile 1 was used as the reference group. All VIF values were less than 5, indicating no serious multicollinearity among the independent variables. The same set of independent variables was included in the adjusted logistic regression models for CAS, LEAD, and DPN.

**Supplementary Table S2. Association between quartile of FIB-4 and DPN/LEAD.**

|  |  | **Q1** | **Q2 OR (95% CI)** | **P** | **Q3 OR (95% CI)** | **P** | **Q4 OR (95% CI)** | **P** |
| --- | --- | --- | --- | --- | --- | --- | --- | --- |
| **DPN** | Model 1 | **1** | 1.47 (0.95–2.28) | 0.085 | 1.28 (0.83–1.97) | 0.265 | 1.44 (0.94–2.20) | 0.095 |
|  | Model 2 | **1** | 1.51 (0.97–2.34) | 0.069 | 1.31 (0.85–2.02) | 0.223 | 1.49 (0.97–2.28) | 0.068 |
|  | Model 3 | **1** | 1.43 (0.91–2.24) | 0.123 | 1.17 (0.75–1.84) | 0.484 | 1.37 (0.88–2.12) | 0.164 |
| **LEAD** | Model 1 | **1** | 1.12 (0.76–1.65) | 0.581 | 1.74 (1.18–2.57) | 0.005 | 2.05 (1.39–3.04) | <0.001 |
|  | Model 2 | **1** | 1.12 (0.75–1.65) | 0.583 | 1.73 (1.17–2.56) | 0.006 | 2.02 (1.36–3.00) | <0.001 |
|  | Model 3 | **1** | 1.09 (0.72–1.63) | 0.688 | 1.60 (1.06–2.41) | 0.024 | 1.88 (1.24–2.84) | 0.003 |

Results are expressed as OR (95% CI) from binary logistic regression. Q1 is the reference group. Model 1: unadjusted. Model 2: adjusted for sex, smoking, and alcohol consumption. Model 3: adjusted for sex, smoking, alcohol consumption, BMI, FBG, GGT, HbA1c, TG, SUA, Cr, and hypertension.
Abbreviations: DPN, diabetic sensorimotor polyneuropathy; LEAD, lower extremity arterial disease; BMI, body mass index; FBG, fasting blood glucose; GGT, glutamyl transpeptidase; HbA1c, glycated hemoglobin A1c; TG, triglyceride; SUA, serum uric acid; Cr, creatinine.

**Supplementary Table S3. Diagnostic performance of FIB-4 for DPN and LEAD.**

| **Outcome** | **AUC (95% CI)** | **Cut-off value** | **Sensitivity (%)** | **Specificity (%)** | **PPV (%)** | **NPV (%)** | **Youden index** |
| --- | --- | --- | --- | --- | --- | --- | --- |
| DPN | 0.532 (0.492–0.568) | 0.72 | 78.9 | 29.2 | 29.5 | 78.7 | 0.082 |
| LEAD | 0.587 (0.552–0.626) | 0.91 | 64.8 | 51.1 | 74.4 | 39.8 | 0.158 |

Optimal cut-off value was determined by the maximum Youden index. AUC 95% CI derived by 1,000-iteration bootstrap resampling.
Abbreviations: AUC, area under the receiver operating characteristic curve; PPV, positive predictive value; NPV, negative predictive value; DPN, diabetic sensorimotor polyneuropathy; LEAD, lower extremity arterial disease.

**Supplementary Figure S1*.* The RCS curve of the association between FIB-4 and DPN, LEAD odds ratio among all the study participants.**
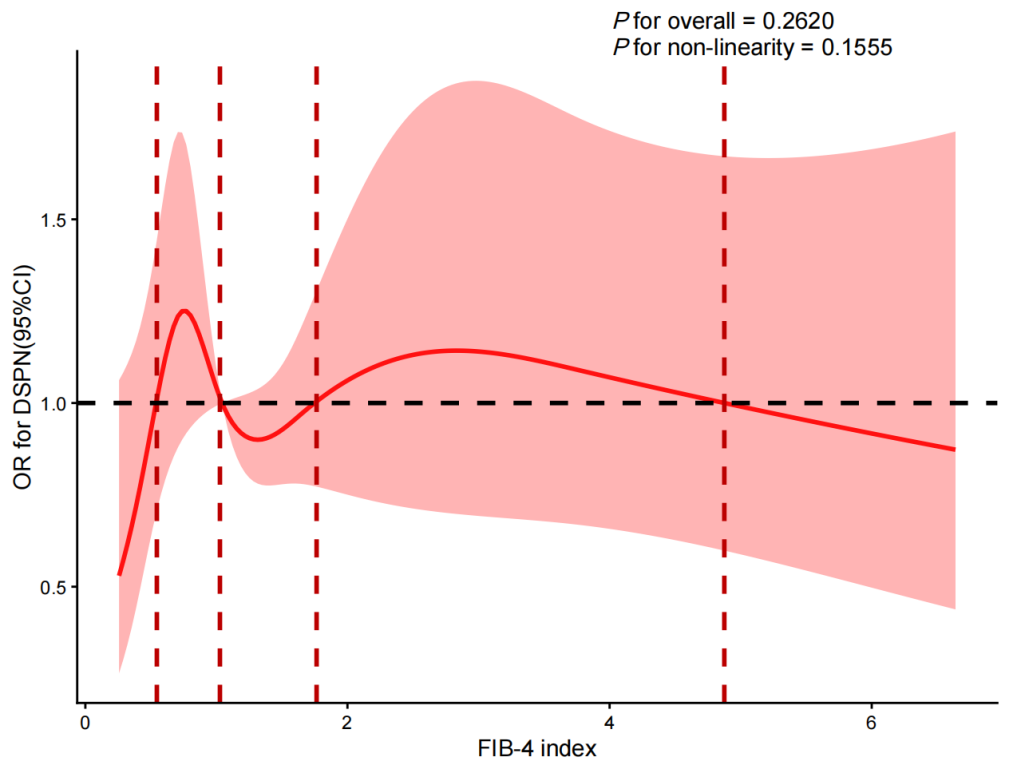

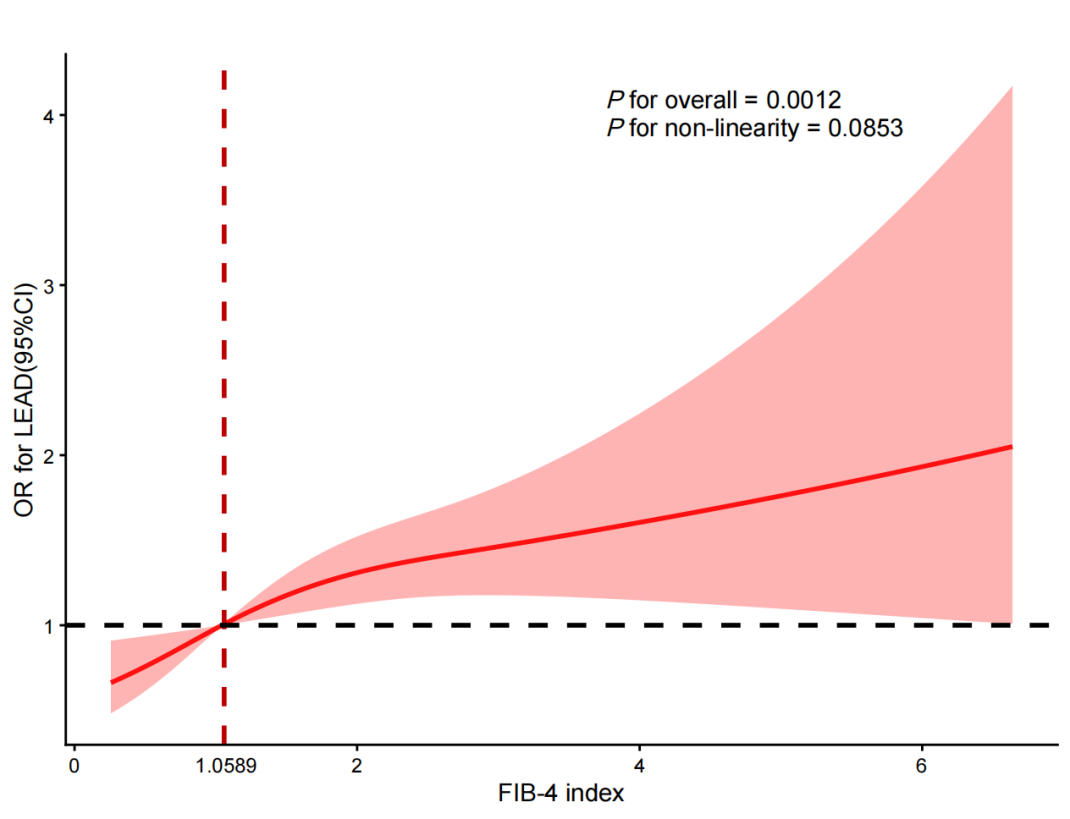


CI: confidence interval. RCS, restricted cubic spline.

**Supplementary Figure S2. Diagnostic performance of FIB-4 for DPN and LEAD**


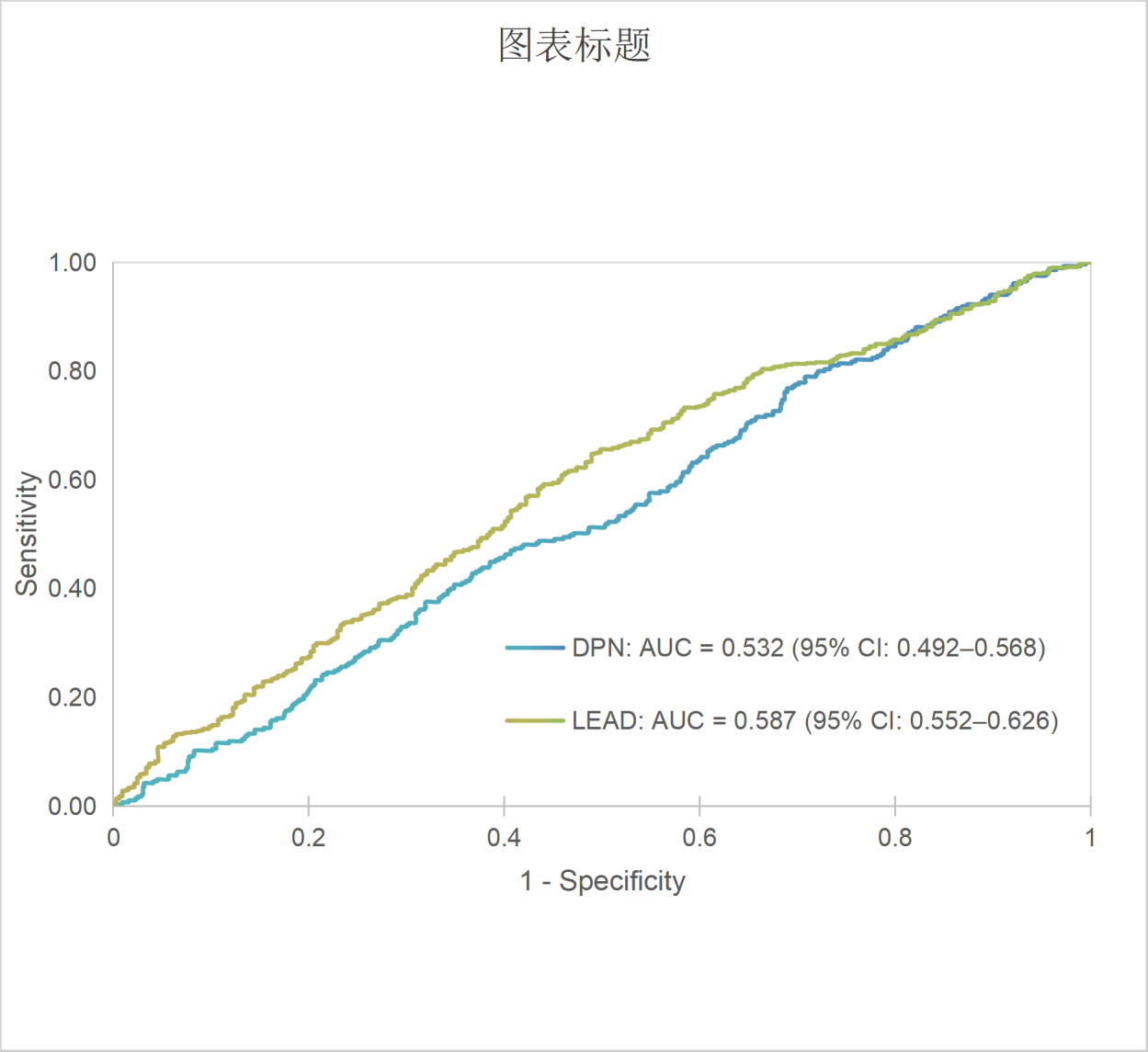


**Supplementary Figure S3. Subgroup analyses of the association between FIB-4 and LEAD.**

###

Note 1: The above model adjusted for sex/smoking/drinking/BMI/FBG/GGT/HbA1c/TG/SUA/Cr/HN. Note 2: In each case, the model is not adjusted for the stratification variable. Note 3: Given the multiple subgroup analyses, we acknowledge the potential for Type I errors. After Bonferroni correction, significant associations remained in but not in other subgroups.

**Supplementary Figure S4. Subgroup analyses of the association between FIB-4 and DPN**

Note 1: The above model adjusted for sex/smoking/drinking/BMI/FBG/GGT/HbA1c/TG/SUA/Cr/HN. Note 2: In each case, the model is not adjusted for the stratification variable. Note 3: Given the multiple subgroup analyses, we acknowledge the potential for Type I errors. After Bonferroni correction, significant associations remained in but not in other subgroups.
